# Roles of the Chemokine Receptor CX3CR1 in the Pathogenesis of RSV Infections

**DOI:** 10.3390/v18040463

**Published:** 2026-04-13

**Authors:** Robert Meineke, Martin Ludlow, Albert D. M. E. Osterhaus, Guus F. Rimmelzwaan

**Affiliations:** Research Center for Emerging Infections and Zoonoses, University of Veterinary Medicine Hannover, 30559 Hannover, Germany

**Keywords:** RSV, glycoprotein, CX3CR1, immunity, immunopathology

## Abstract

CX3CR1 is a chemokine receptor expressed on respiratory epithelial and immune cells and has been identified as a host factor important for infections with respiratory syncytial virus (RSV). In this review, we discuss the roles CX3CR1 plays in the pathogenesis of RSV infections as a viral entry receptor and regulator of immune cell trafficking. The conserved CX3C motif of the RSV G glycoprotein binds to CX3CR1 to mediate viral attachment and entry into respiratory epithelial cells. Furthermore, soluble G protein (sG) can bind to CX3CR1 and competitively interfere with cell signaling induced by the chemokine CX3CL1, resulting in inhibition of immune cell recruitment to the site of infection. In addition, sG engages TLR2 on epithelial cells, activating MyD88-NF-κB signaling and priming the NLRP3 inflammasome, which enhances viral dissemination through pyroptotic cell death. CX3CR1 signaling should be viewed as one of several overlapping host factors that—along with developmental changes in interferon and STAT3 signaling, airway anatomy, inflammasome activity, and tissue-resident memory responses—contribute to differential disease outcomes of RSV infection. A more complete molecular understanding of RSV-CX3CR1 interactions and downstream host responses may enable the development of improved prevention and treatment strategies.

## 1. Introduction

Respiratory syncytial virus (RSV) causes approximately 33 million lower respiratory tract infections and more than 100,000 deaths each year in children under five years of age [1,2]. Infants, older adults, and immunocompromised individuals carry a disproportionate burden of severe disease and mortality [3,4,5,6,7,8]. The age-defined risk groups display various clinical manifestations upon RSV infection, ranging from life-threatening bronchiolitis in young infants to exacerbations of chronic cardiopulmonary conditions in older adults.

During the first months of life, infants benefit from maternally derived antibodies transferred across the placenta, but RSV-specific titers decline rapidly [9,10,11]. This waning of passively derived antibody levels coincides with a sharp increase in the incidence of RSV-related hospitalizations from 17 per 1000 births overall in the United States, increasing to 30 per 1000 in high-risk populations, with the peak incidence occurring at 2–3 months of age [12,13,14]. At this age, infants become susceptible to RSV infection, and their innate and adaptive immune systems are still immature. Because of the small diameter of their airways, even modest inflammation can result in critical airflow restriction.

Upon RSV infection, virus-specific antibodies and T-cell responses are induced, which contribute to protective immunity [15,16]. Compared to adults, infants mount delayed and lower virus-specific CD8+ T-cell responses. In older adults, the rapid expansion of memory (tissue-resident) T cells can restrict viral replication but may aggravate airway inflammation upon reinfection [17,18,19,20]. Novel insights into the structural conformation of the RSV F protein have enabled the development of effective vaccines and protective antibodies. Antibodies that recognize the prefusion conformation of the F protein have substantially more virus-neutralizing activity than those that bind to postfusion F, and prefusion F-based vaccines have now been licensed for maternal immunization and for use in older adults [21,22]. Clinical trials have demonstrated robust protection in these populations, but the early termination of a pediatric mRNA vaccine trial in 2024 because of a safety signal has renewed concern about age-dependent immune responses to RSV vaccination in very young infants, and highlights that mechanisms remain incompletely understood [23,24]. Together with the emergence of RSV strains resistant to the F-targeting monoclonal antibody nirsevimab [25], these observations highlight the need to better understand alternative viral targets and age-dependent host factors that define the risk for severe disease and vaccine responses.

The chemokine receptor CX3CR1 has emerged as one of the components that may contribute to the differences in clinical manifestations of RSV infections. CX3CR1 is expressed on respiratory epithelial cells and multiple immune cell subsets and is involved in cell trafficking, tissue surveillance, and maintenance of immune homeostasis [26,27,28,29]. RSV can use CX3CR1 as one of its receptors by binding the G glycoprotein anchored in the viral envelope through its conserved CX3C motif, which facilitates viral entry.

Importantly, CX3CR1 expression is not the sole host factor contributing to differences in the clinical outcome of RSV infections. Other factors include interferon and STAT3 signaling, airway anatomy, inflammasome activity, and tissue-resident memory responses [30,31,32,33,34,35,36,37,38,39]. CX3CR1 thus plays distinct roles in RSV pathogenesis that warrant detailed review. As mentioned above, CX3CR1 serves as an entry receptor for RSV through binding of the G glycoprotein in its envelope, initiating viral infection. Furthermore, CX3CR1 signaling mediates the recruitment of immune cells to sites of infection through its natural ligand CX3CL1 (fractalkine), contributing to protective antiviral responses. During RSV infection, a soluble form (sG) is released from infected cells [40,41], which is generated by alternative translation initiation at the second AUG codon corresponding to amino acid position 48 (M48). Soluble G has a structural resemblance to the chemokine CX3CL1 and acts as a competitive antagonist of CX3CL1, inhibiting immune cell trafficking [2,34,42,43,44,45,46,47,48]. Recent evidence has demonstrated that sG also engages Toll-like receptor 2 (TLR2), activating proinflammatory signaling and priming the NLRP3 inflammasome for enhanced viral dissemination [49]. In the present review, we discuss how CX3CR1 expression and function in epithelial and immune cells influence the pathogenesis of RSV infections, with a particular focus on the immunomodulatory properties of the G protein.

## 2. Age-Dependent CX3CR1 Expression

CX3CR1 expression patterns change during the human lifespan, which may have an impact on the cellular microenvironment of the respiratory tract and susceptibility to RSV infection [34,50,51]. Airway epithelial cells in nasopharyngeal, tracheal, and bronchial passages express CX3CR1 and are susceptible to infections with RSV [52]. After birth, airway epithelial differentiation leads to increased CX3CR1 expression specifically on ciliated columnar epithelial cells, which are primary targets for RSV infection [34,45,52].

Lung resident immune cells also express CX3CR1, but the extent of expression is age-dependent. Alveolar macrophages, dendritic cells, and tissue-resident CD8+ T lymphocytes express CX3CR1, with expression levels differing substantially across cell types and maturation states [53,54,55]. In older adults, senescent immune cells display altered CX3CR1 expression or responsiveness to its ligand CX3CL1 [51,56,57,58]. Thus, in older adults, reduced CX3CR1 signaling may correlate with poor disease outcome of RSV infections.

The mechanisms underlying age-dependent CX3CR1 expression in the human airway are not yet fully defined. One likely contributor is postnatal epithelial maturation, since epithelial differentiation increases the abundance of ciliated cells, the airway cell population in which CX3CR1 expression is most abundantly detected [34,52]. In immune cells, CX3CR1 expression can be modulated by cytokine-responsive pathways, including interferon- and PI3K/Akt-dependent signaling [59,60,61], suggesting that developmental differences in JAK/STAT signaling or receptor turnover may influence age-dependent responsiveness during RSV infection. However, direct evidence linking these regulatory mechanisms to CX3CR1 expression across age groups in human RSV infection remains limited.

**Figure 1:**
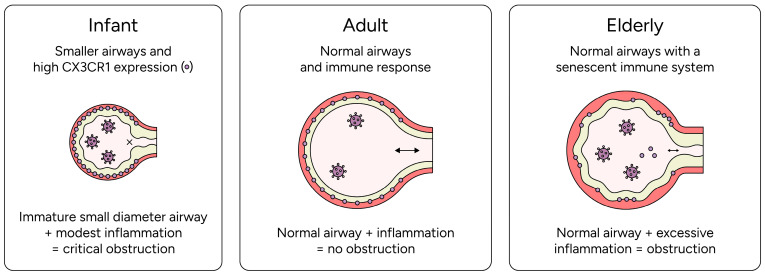
**Age-dependent CX3CR1 expression and airway geometry as potential modifiers of RSV disease severity.** Infant airways may exhibit higher CX3CR1 expression on ciliated epithelium and have a small luminal diameter that is anatomically prone to clinically relevant obstruction from inflammation and mucus accumulation (yellow) (**left**). Adult airways show balanced CX3CR1-associated immune recruitment with a lower risk of critical luminal compromise under comparable inflammatory conditions (**middle**). Elderly airways may display altered CX3CR1 signaling together with epithelial senescence and heightened inflammatory susceptibility, which could promote moderate airflow limitation despite preserved airway geometry (**right**).

In addition to inflammatory processes, including those mediated by CX3CR1 signaling, age-dependent differences in airway anatomy shape the clinical manifestation of RSV infections [62,63]. Compared to older children and adults, the airways of infants have significantly smaller diameters, narrower conducting passages, and less developed collateral ventilation pathways. Airway resistance follows the Hagen–Poiseuille relationship and is inversely proportional to the fourth power of the airway radius [62,64]. Consequently, a similar degree of mucosal edema, mucus accumulation, and epithelial debris can cause disproportionately greater airflow obstruction in infants than in older individuals [65] (Figure 1). CX3CR1-dependent inflammatory pathways may therefore have greater clinical consequences in infants, but their quantitative contribution to airflow obstruction has not been defined.

## 3. Molecular Interactions Between CX3CR1 and the G Protein

The RSV G glycoprotein is a heavily glycosylated type II transmembrane protein that spans 289–299 amino acids, depending on the strain [2,47,66]. Its central conserved region contains a CX3C chemokine motif (amino acids 182–186, sequence CWAIC) that structurally mimics the ligand of CX3CR1, the chemokine CX3CL1 [46]. The CX3C motif of the RSV G protein mediates high-affinity binding to CX3CR1 on airway epithelial cells and immune cell populations, facilitating viral attachment in addition to other receptors, such as nucleolin and glycosaminoglycans [2,40,46,47,66]. Mutagenesis studies confirmed that the motif is an important determinant of CX3CR1-dependent infection: CX3C-to-CX4C mutations disrupted receptor binding and substantially reduced the infection of primary human airway epithelial cells [52,66,67,68]. However, RSV can also use alternative attachment factors [69,70,71,72], and the CX3C motif should therefore not be interpreted as the sole determinant of cellular entry.

The CX3C motif is highly conserved across the RSV A and B subtypes, with greater than 95% identity in the central conserved domain [66,73]. Monoclonal antibodies that block G protein–CX3CR1 interactions cross-neutralize strains from both subtypes. In marked contrast, the entire G protein sequence ranks among the most variable of all known viral proteins, with extensive hypervariability in the mucin-like regions across strains and seasons. This pattern suggests that immune evasion pressure drives G protein diversity, whereas conservation of the CX3C motif may reflect functional constraints. This distinction has direct implications for vaccine design, since conservation of the motif supports the idea that CX3CR1-related G functions remain biologically important across strains, even if they are not the sole determinants of infection.

Computational modeling provided mechanistic insights into the interaction of RSV G and CX3CR1 [74]. Hydrophobic patches flanking the CX3C motif contact the extracellular loops of CX3CR1, optimally positioning the chemokine motif for receptor engagement. These predictions await experimental validation through quantitative binding kinetics, X-ray crystallography, or cryo-electron microscopy of the G protein–CX3CR1 complex. However, even confirmed high-affinity binding would not determine the contribution of CX3CR1 binding to infection efficiency relative to binding to other receptors.

Membrane-bound G protein engages CX3CR1 to mediate viral entry and activate intracellular signaling cascades like phosphoinositide 3-kinase (PI3K), phosphoinositide-dependent kinase 1 (PDK1), Akt (PKB), and IκB kinase (IKK) [42,45,52,75]. Other attachment- or entry-associated interactions have been reported for RSV, including binding to glycosaminoglycans and alternative receptor candidates [69,70,71,72]. RSV infection through CX3CR1 or parallel pathways induces increased nucleolin expression to facilitate replication, together with decreased expression of ciliary function genes (CC2D2A, CFAP221) and tight junction proteins that compromise epithelial barrier integrity [75,76]. The sole CX3CR1 ligand, the chemokine CX3CL1, generally activates the PI3K/Akt, ERK/MAPK, and NF-κB pathways, with downstream effects on cell survival, differentiation, and inflammation [77,78,79,80].

Epidemiological studies examining associations between G protein sequence variation and disease severity have yielded conflicting results, with most associations disappearing after adjustment for viral load, age, and comorbidities [81,82,83]. For the purposes of this review, the critical observation is that the CX3C motif is highly conserved across RSV strains despite extensive variation elsewhere in the G protein [66,73,84], suggesting that CX3CR1-dependent functions remain under functional constraint even as immune evasion drives broader G-protein evolution. Whether non-conserved G sequences influence disease severity through mechanisms independent of CX3CR1 remains an open question and is outside the scope of this review.

Apart from being a receptor-binding protein important for viral entry, the RSV G protein also has immunomodulatory properties both as a membrane-bound and soluble form [85]. The soluble form (sG protein) is produced by RSV-infected cells through alternative translation initiation at the second AUG codon corresponding to M48 [2,46,86]. This soluble form was believed to serve as an immunological decoy for virus-neutralizing antibodies directed to the G protein, but recently, it was found that sG has various immunomodulatory properties that contribute to viral pathogenesis through mechanisms independent of viral entry (see below).

## 4. Immunomodulatory Functions of the RSV G Protein

**Figure 2:**
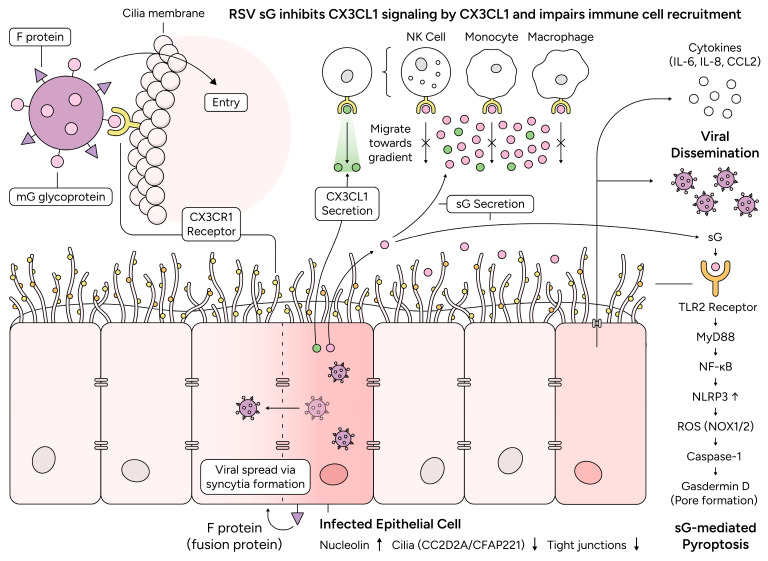
**Dual role of RSV G protein in exploiting CX3CR1 for viral entry and immune evasion.** Membrane-bound G glycoprotein (pink circle on virion) binds CX3CR1 on epithelial cells to mediate viral attachment/entry, while soluble G (sG, pink circle) competitively inhibits CX3CL1 signaling, impairing NK cell, monocyte, and macrophage recruitment. sG simultaneously engages TLR2 (orange), activating MyD88-NF-κB and priming NLRP3 inflammasome (NLRP3, NOX1/2, ROS) for caspase-1-/GSDMD-mediated pyroptosis, enhancing viral dissemination and secretion of IL-6, IL-8, and CCL2. The mechanistic relationship between CX3CR1 binding and TLR2 engagement remains to be defined.

The RSV G protein exerts immunomodulatory effects through multiple independent mechanisms that collectively impair antiviral immunity and promote viral dissemination. These mechanisms operate through both CX3CR1-dependent and CX3CR1-independent pathways, with the soluble form (sG) playing a central role in paracrine immune modulation (Figure 2).

sG acts as a competitive antagonist of the CX3CR1 ligand CX3CL1 (fractalkine), thereby potentially impairing the recruitment of CX3CR1-expressing leukocyte populations, including NK cells, monocytes, macrophages, and subsets of T cells, to sites of RSV infection. We recently demonstrated that recombinant RSV sG can bind to CX3CR1 without activating receptor signaling [46]. Instead, sG competitively inhibits CX3CL1-induced calcium flux in a dose-dependent manner, with half-maximal inhibition occurring at equimolar sG:CX3CL1 ratios [46]. This antagonism impaired monocyte migration and adhesion in vitro, which are requirements for efficient immune cell recruitment to sites of RSV infection [46]. The extent of inhibition was dependent on the soluble G concentration relative to that of CX3CL1, their binding affinities, receptor occupancy kinetics, and the threshold occupancy required to block CX3CL1-mediated responses [46,87]. Information on RSV sG and CX3CL1 concentrations in bronchoalveolar lavage fluid during acute human RSV infection is scarce. Although CX3CL1 levels are elevated in chronic inflammatory lung diseases [88,89], measurements of soluble G, CX3CL1, and their ratio during acute RSV are lacking. Determining whether soluble G inhibits overall immune recruitment requires the concurrent assessment of parallel chemokine pathways (CCL2, CXCL10, and IL-8) that redundantly drive leukocyte trafficking to RSV infection sites [90,91].

In addition to antagonizing CX3CR1 activation by CX3CL1, sG can engage Toll-like receptor 2 (TLR2) on airway epithelial cells, activating a distinct immunomodulatory pathway [92]. We demonstrated that recombinant sG can bind TLR2 and activate MyD88-NF-κB signaling, leading to the secretion of proinflammatory mediators, including IL-6, IL-8, VEGF, and CCL2 [49]. This TLR2-mediated signaling provided the priming signal for NLRP3 inflammasome activation by upregulating NLRP3 expression and inducing reactive oxygen species (ROS) generation through NADPH oxidase (NOX1/NOX2) activation [49]. Pretreatment of airway epithelial cells with RSV sG substantially enhanced viral replication upon subsequent RSV infection, demonstrating that sG functions beyond canonical receptor binding [49]. Importantly, sG-primed cells exhibited markedly increased caspase-1 activation and gasdermin D (GSDMD) cleavage upon RSV infection, leading to pyroptotic cell death [49]. This pyroptosis can facilitate viral egress and dissemination while causing inflammatory tissue damage [49]. The TLR2-NLRP3 axis thus represents a paracrine mechanism through which sG released from infected cells primes neighboring uninfected cells for enhanced inflammasome activation upon subsequent viral encounter, increasing viral spread and immunopathology.

Current data support two separable activities of RSV sG. First, sG binds CX3CR1 and antagonizes CX3CL1-dependent signaling without inducing canonical CX3CR1 activation [46]. Second, sG can engage TLR2 on airway epithelial cells and activate MyD88-NF-kB signaling that primes NLRP3 activation [49]. Whether CX3CR1 binding facilitates TLR2 engagement, alters ligand presentation at the cell surface, or occurs in parallel as a mechanistically independent activity has not yet been resolved experimentally. We therefore consider these as distinct pathways that may converge at the level of local inflammation rather than as a defined linear signaling hierarchy.

RSV-infected epithelial cells release inflammatory mediators, including IL-6, IL-8, and CXCL10, which further increase local inflammation and the recruitment of immune cells [93]. These pathways operate in parallel with CX3CR1-mediated mechanisms, and their relative contributions are largely unknown. In addition to effects on CX3CR1 signaling, G protein glycosylation patterns influence Th1/Th2 cytokine balance through pattern recognition receptors. The glycosylated G protein produced in mammalian cells enhanced lung pathology and Th2 cytokine production (IL-4, IL-5, and IL-13) upon infection in mice, whereas nonglycosylated RSV G produced in bacterial expression systems afforded protection without causing pathology [94]. This highlights the glycosylation dependency of the immunomodulatory capacity of RSV glycoproteins, which occurs through modulation of TLR2-, TLR4-, and CX3CR1-related pathways [46,49,92,95], with soluble G further augmenting IL-5 production and eosinophil recruitment in preclinical models [96]. Thus, the G protein drives disease severity through multiple independent mechanisms affecting both innate recruitment and Th1/Th2 balance.

## 5. Innate Immune Response and Dependency on CX3CR1 Signaling

**Figure 3:**
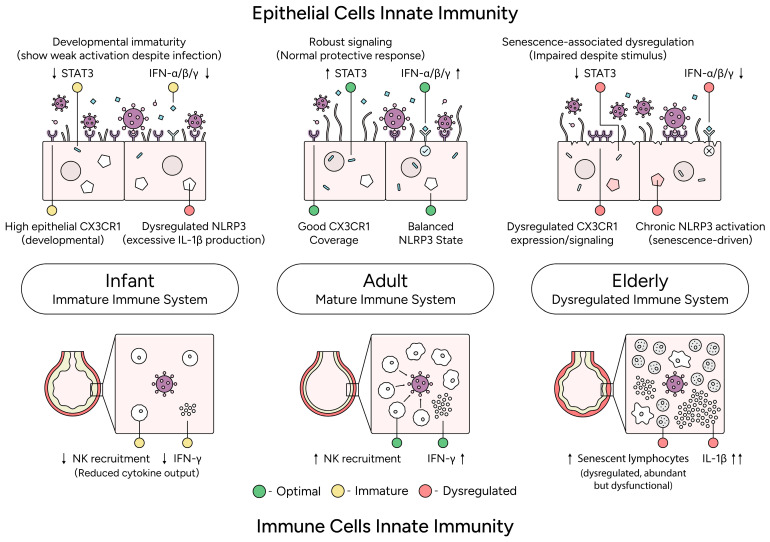
**Age-stratified innate immune dysregulation via CX3CR1 signaling.** Top panel (epithelial cells): Infants exhibit weak STAT3 and IFN-α/β/γ responses, impairing antiviral state; adults show robust signaling; elderly display senescence-associated reduced STAT3/IFN-α/β/γ. Bottom panel (immune cells): Infants have impaired NK recruitment/IFN-γ production; adults demonstrate optimal recruitment; elderly feature senescent lymphocytes with increased NLRP3/IL-1β inflammasome hyperactivity.

Age-dependent differences in innate immunity to RSV involve multiple overlapping mechanisms that converge with CX3CR1 signaling (Figure 3). Type I interferons (IFN-α/β) provide critical early viral control, but neonatal epithelial cells generate substantially lower responses than adult cells owing to incomplete maturation of IRF3, IRF7, RIG-I, and MDA5 [33,97]. Type I interferons regulate CX3CL1 expression, and type II interferons upregulate CX3CR1 expression on immune cells via PI3K/Akt signaling [59,60,61,98], so blunted neonatal interferon responses may reduce both CX3CL1 availability and CX3CR1 expression independent of RSV-mediated sG antagonism [46]. Similarly, STAT3 signaling supports epithelial survival in adults but is impaired in neonates [36,99], and RSV infection can dysregulate ciliary genes, including CC2D2A and CFAP221, further compromising epithelial defenses [75]. These age-dependent differences in interferon and STAT signaling create a permissive microenvironment in which CX3CR1-mediated immune recruitment may be further impaired by RSV sG.

CX3CR1-expressing NK cells mediate early viral clearance through direct cytotoxicity and IFN-γ production, and CX3CR1-deficient mice show impaired NK trafficking and viral control [100,101,102]. Neonatal NK cells are developmentally immature and reduced in number [103,104], and their CX3CR1-mediated recruitment to the respiratory tract is likely further compromised by RSV sG antagonism [46]. However, NK cell immaturity is not solely a consequence of reduced CX3CR1 signaling, and it reflects a broader developmental constraint in the neonatal innate immune system.

Neonatal alveolar macrophages exhibit inherent dysfunction independent of CX3CR1: they mount delayed and weak responses to RSV with limited upregulation of MHC class II, CD86, TNF-α, IL-6, and IL-1β, whereas adult macrophages show robust classical activation [105,106]. Restoration of classical function in neonatal macrophages by exogenous IFN-γ demonstrates that this age-dependent difference reflects developmental signaling constraints rather than simply impaired trafficking [105]. Nevertheless, reduced CX3CR1-mediated recruitment by RSV sG-mediated antagonism may still exacerbate macrophage dysfunction by delaying the arrival of innate immune cells to infection sites.

Animal models reveal age-specific CX3CR1 functions: adult CX3CR1-deficient mice show impaired NK and neutrophil trafficking, reduced antiviral cytokine production, and compromised viral control [100,107], whereas neonatal CX3CR1-deficient mice develop exaggerated IL-17-driven neutrophilic inflammation yet maintain intact viral clearance [107,108]. This divergence suggests that CX3CR1 shifts from supporting protective effector-cell recruitment in adults to restraining excessive innate inflammation in neonates. Importantly, CX3CR1 genetic deletion likely overestimates therapeutic blockade effects, because long-term knockout permits compensatory upregulation of parallel chemokine pathways (CCR5, CXCR1-2, and CXCR3), whereas acute sG-mediated receptor antagonism occurs without compensation [46,109]. The distinct IL-17-neutrophilic (rather than Th2-eosinophilic) pathology in neonatal CX3CR1-deficient mice also differs from FI-RSV vaccine-enhanced disease, suggesting that genetic knockout models may not faithfully predict therapeutic CX3CR1 modulation during natural infection [107,108,109,110,111].

## 6. Vaccine-Enhanced Disease and Potential CX3CR1-Related Modifiers

The formalin-inactivated RSV (FI-RSV) vaccine trials of 1966–1967 represent a defining cautionary episode in the history of the development of RSV vaccines [112,113]. Administration of formalin-inactivated whole-virion RSV preparation (FI-RSV) adjuvanted with alum predisposed infants to enhanced disease upon natural RSV infection [112,113]. Vaccine-enhanced disease was characterized by severe lower respiratory infection, associated with airway inflammation and eosinophilia and sometimes had a fatal outcome [112,113].

Various mechanisms may underlie the predisposition for this vaccine-enhanced disease [114]. Formalin inactivation disrupts the prefusion conformation of the F protein while preserving postfusion forms, resulting in the induction of low-avidity non-neutralizing antibodies that form pathogenic immune complexes upon viral infection [115,116]. These complexes activate complement through Fcγ receptor engagement, generating C3a and C5a anaphylatoxins that trigger mast cell degranulation and airway obstruction [116]. This process was not dependent on the presence of the G protein, as FI-RSV without the G protein still resulted in vaccine-enhanced disease. The dependence of FcγR expression was confirmed in FcγR-deficient mice, which proved to be protected from challenge infection rather than displaying enhanced disease. [117,118]. In contrast, other studies have shown that the G protein could amplify F-mediated enhanced disease by alternative mechanisms [96,119].

In FI-RSV-vaccinated mice challenged with WT RSV, Th2 cytokines (IL-4, IL-5, IL-13) drive eosinophil recruitment [110,120]. FI-RSV lacking G induced 50–60% less eosinophilia than WT FI-RSV [117,121], showing that G recognition further amplifies Th2-driven eosinophilic pathology in addition to the F protein- and immune-complex-dependent mechanisms described above. CX3CR1 blockade or challenge with RSV bearing CX3C mutations further reduced eosinophilia and pathology compared to untreated control or challenge with the wild-type virus, respectively [111,122], indicating that G protein engagement of CX3CR1 acts as a modifier of vaccine-enhanced disease severity rather than as its primary cause. In addition, CD4+ T cells contributed to the enhancement of disease by Th2 cytokine production. In mice, FI-RSV primed excessive IL-4, IL-5, and IL-13 production, which promotes eosinophil infiltration, mucus production, and airway hyperreactivity upon RSV challenge [110,120,123]. Taken together, these data support a model in which FI-RSV-enhanced disease is initiated by F protein- and immune-complex-driven mechanisms, with G protein–CX3CR1 interactions providing an additional layer of Th2-biased amplification.

Neither eosinophil depletion nor STAT6 knockout in mice completely prevented vaccine-enhanced disease, which underscores the importance of the immune complex–complement axis to provide inflammatory priming that is amplified by Th2 responses [123,124]. Additionally, intramuscular immunization with FI-RSV failed to induce mucosal IgA and generated poor CD8+ T-cell responses, unlike live RSV infection [116,125,126].

Thus, FI-RSV vaccination predisposes infants to enhanced disease severity through pathogenic vaccine-induced immunity, which already has developmental immune constraints [33,34,119]. The enhanced disease predisposed by vaccination of infants is most likely amplified by Th2-biased immune responses in this age group [127,128]. Vaccinated infants exhibited a combined Th2 bias [110], immune complex activation [116], and G protein-mediated TLR2/CX3CR1 amplification [46,49,95,111], compounded by reduced NK cytotoxicity [103], alternatively activated macrophages [105], impaired STAT3 protection [36], and IRF3/7 immaturity [97]. Comparable vaccine-enhanced disease was not observed in previously RSV-exposed adults who received FI-RSV, highlighting age and immune maturation as important determinants of FI-RSV safety.

Modern vaccine design has addressed the key mechanisms underlying FI-RSV-associated disease. Prefusion F protein-based vaccines (Arexvy, mRESVIA, and Abrysvo) have received approval for use in older adults and maternal immunization, with the metastable prefusion conformation eliciting high-avidity neutralizing antibodies that prevent immune complex formation [129,130,131]. Various studies in older adults demonstrated approximately 80–90% vaccine effectiveness against lower respiratory hospitalization without evidence of vaccine-enhanced respiratory disease [132,133,134,135]. However, a pediatric mRNA RSV vaccine trial was halted after vaccinated infants developed severe lower respiratory infections upon natural challenge at higher frequencies than recipients who received a placebo [23,24]. These findings underscore that age-dependent features of the infant immune system can influence vaccine outcomes in ways that remain incompletely understood.

## 7. Implications for the Development of Future Intervention Strategies

CX3CR1 plays various roles in the pathogenesis of RSV infections. First, CX3CR1 serves as one of the receptors for RSV, which the virus can bind to through the CX3C motif of the G protein, which is one of the envelope proteins. For this reason, the G protein has been considered an attractive target for vaccine design, since the induction of antibodies directed to the CX3C motif could prevent the virus from binding to its receptor [38,66,136]. However, the G ectodomain is highly variable across circulating strains and heavily O-glycosylated, which can mask conserved epitopes and limit the breadth of antibody responses. These features contributed to the decision to favor prefusion F as the principal antigen for licensed RSV vaccines. However, passive administration of CX3C motif-specific antibodies may offer a certain degree of protection against infection [67,136,137].

However, the CX3CR1 receptor plays alternative roles in the pathogenesis of RSV infections, and the interaction with the viral G protein, either as an envelope or as a soluble protein, may have a profound effect on the clinical outcome of RSV infections. We have demonstrated that the RSV sG protein can structurally mimic the natural ligand of CX3CR1, the chemokine CX3CL1, and competitively inhibit the function of CX3CL1 by binding to CX3CR1. These ultimately result in the inhibition of the recruitment of immune cells to the site of infection. Viral chemokine mimicry is more commonly described for herpesviruses than for respiratory viruses [138,139]. However, the CX3C motif is highly conserved across RSV strains despite the hypervariability of the G protein, suggesting strong functional constraints [66,84,140]. The acquisition of antibodies to the CX3C motif, either passively or actively by vaccination, may offer a second mode of action in addition to their virus-neutralizing activity [38,66,67,136,137,141]. These antibodies may prevent the inhibition of CX3CL1-mediated CX3CR1 signaling and thus have a beneficial effect on the course of infection by improving the recruitment of immune cells to the site of infection. Given the sequence variability and glycosylation of G, such G-directed approaches are most likely to be effective when they focus on conserved functional regions and are used in combination with established prefusion F-based strategies rather than as stand-alone replacements.

Lastly, we have identified yet another mode of action of the G protein, in which the interaction with CX3CR1 and the subsequent engagement and activation of TLR2 play a role. The sG-mediated activation of TLR2 leads to NLRP3 inflammasome signaling and subsequently to pyroptosis, which favors viral dissemination. Additionally, this activity of the G protein may be inhibited by specific antibodies acquired by vaccination or passive transfer. The identification of G-mediated signaling pathways that favor virus replication may open novel avenues for the treatment of RSV infections. However, direct pharmacologic targeting of TLR2, the NLRP3 inflammasome, or pyroptosis must be approached with caution, as these pathways also contribute to protective innate immunity. Any use of such inhibitors would require careful definition of dose, timing, and patient populations to avoid impairing antiviral defense.

Collectively, CX3CR1 plays an important role in the pathogenesis of RSV infections. Of interest, infants, who are at the highest risk for RSV infections, display higher levels of CX3CR1 expression in airway epithelial cells than older individuals. CX3CR1 is not only a viral attachment receptor; its natural function as a chemokine receptor can be subverted by the viral G protein. Furthermore, sequestering RSV sG by CX3CR1 allows sG to engage and activate TLR2, resulting in inflammasome NLRP3 activation and pyroptosis. These findings indicate that sG may be an attractive target for CX3C motif-specific antibodies, either induced by vaccination or administered passively. These antibodies may have virus-neutralizing properties or inhibit the biological activities of sG described above. Alternatively, inhibitors of downstream TLR2 activation may alleviate symptoms and improve the clinical outcome of RSV infections. Further research is warranted to explore these possibilities.

## Data Availability

No new data were created or analyzed in this study.
